# Determination of anti-SARS-CoV-2 virustatic pharmaceuticals in the aquatic environment using high-performance liquid chromatography high-resolution mass spectrometry

**DOI:** 10.1007/s00216-023-04811-7

**Published:** 2023-07-13

**Authors:** Indra Bartels, Martin Jaeger, Torsten C. Schmidt

**Affiliations:** 1 Department of Chemistry and ILOC, Niederrhein University of Applied Sciences, Frankenring 20, 47798 Krefeld, Germany; 2 Faculty of Chemistry, University Duisburg-Essen, Universitätsstraße 5, 45141 Essen, Germany

**Keywords:** HPLC-HRMS, Solid phase extraction, Wastewater treatment, pH stability, Ozonation, Transformation products

## Abstract

The Covid-19 pandemic has affected the global population since 2019. The rapid development and approval of vaccines has brought relief. Yet, effective cures are still being researched. Even if the pandemic situation may end, SARS-CoV-2 will remain and, thus, continued application of the drugs will lead to emissions of the active ingredients into the aquatic environment, as with other anthropogenic micropollutants. However, a general method for trace analysis of antiviral drugs is still missing. To this purpose, favipiravir, remdesivir, its active metabolite GS-441524, molnupiravir and its active metabolite EIDD-1931 were selected as representative analytes. A method was developed based on solid phase extraction and high-performance liquid chromatography combined with electrospray ionization quadrupole time-of-flight high-resolution mass spectrometry. Optimization comprised the choice of chromatographic columns, elution gradient, mass spectrometry and tandem mass spectrometry parameters. Solid phase extraction proved suitable for increase in limits of detection and quantitation. amelioration of the limits of detection and quantitation. Matrix effects were investigated applying the optimized method to a wastewater sample with added virustatics. All five compounds could be separated with reversed phase chromatography, whereas EIDD-1931 profited from hydrophilic interaction liquid chromatography. The optimized method yielded limits of detection and quantification of 2.1·10^-1^, 6.9·10^-1^ µg·L^-1^ for favipiravir, 1.8·10^-3^, 5.5·10^-3^ µg·L^-1^ for remdesivir, 1.9·10^-3^, 7.6·10^-3^ µg·L^-1^ for GS-441524, 2.9·10^-3^, 8.7·10^-3^ µg·L^-1^ for molnupiravir, and 1.3·10^-1^, 3.8·10^-1^ µg·L^-1^ for EIDD 1931. The method was first applied to compound stability testing at pH 2.8 and 9.7. At pH 2.8, remdesivir, GS-441524 and molnupiravir proved stable, whereas about 14% of EIDD-1931 and favipiravir were degraded. All five antiviral compounds were almost completely decomposed at pH 9.7. The application of the method was further demonstrated for potential transformation product detection on favipiravir ozonation monitoring.

**Graphical abstract:**

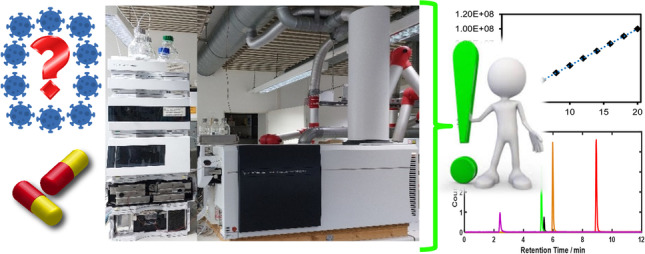

**Supplementary Information:**

The online version contains supplementary material available at 10.1007/s00216-023-04811-7.

## Introduction

During the Covid-19 pandemic anti-SARS-CoV-2 vaccines have been quickly developed and approved for application. Yet, incomplete vaccination of the population, insufficient individual immunization and antibody concentration decreasing with time require small-molecule drugs for cures. At present, four promising antiviral active ingredients are being considered. Their efficacy has already been confirmed [1–3]. Paxlovid combines the active ingredients nirmatrelvir and ritonavir and has been approved in 2022, Lagevrio contains the drug substance molnupiravir (MOL) and Veklury remdesivir (REM) [4, 5]. Both compounds are prodrugs. Their active metabolites are GS-441524 (GS) and EIDD-1931 (EIDD), respectively. Veklury has recently been approved by regulatory agencies, since its application led to a reduction in hospitalization and mortality rates of 87% [6]. In October 2020, the US Food and Drug Administration (FDA) announced the approval of the antiviral pharmaceutical Avigan with the active ingredient favipiravir (FAV) for the treatment of SARS-CoV-2.

Anthropogenic micropollutants, such as pharmaceuticals from households, hospitals or agriculture, are known to cause hazardous effects on aquatic organisms, *e.g.* lethal or motility-inhibiting effects, increasing resistance of microorganisms or inducing feminization of fish [7–9]. A comprehensive detailed overview summarized studies on drug concentrations in the environment. The collected measured environmental concentrations (MECs) data, including antiviral substances detected in Asia and Africa, were transferred to a global database [10]. The antiviral agent oseltamivir was increasingly used against swine flu in 2009 and was detected in the river Rhine [11]. Ritonavir is used for HIV infections and consequently has already been found in various aquatic species worldwide [12, 13]. Hence, the approved and applied anti-SARS-CoV-2 drugs, especially REM, nirmatrelvir and MOL, would be expected to occur in the aquatic environment with increasing frequency, provided the anticipated continuation of the pandemic or endemization [11]. The occurrence of FAV and REM before and after wastewater treatment plants (WWTP) was reported among the elimination and ecotoxicity of fifty-two antiviral agents [14–20]. Especially FAV has been detected in influents, effluents and surface, ground and drinking water [11, 21]. The analytical methods allowing identification and quantitation employed high performance-liquid chromatography (HPLC) in combination with mass spectrometry (MS) as predominant techniques [14, 22–25]. For trace analysis, solid phase extraction (SPE) preceded HPLC-MS methods [26]. In various locations in South Africa, *e*.*g*. in surface waters and WWTP effluent, numerous antiviral agents, such as ritonavir, were found in concentrations of 3·10^-2^ - 1.48·10^3^ ng·L^-1^ [12, 27]. Following regulatory procedures, ecotoxicity assays have not been performed in sufficient quantity to allow ecological hazard assessment [28]. The main focus of the research has of course been set on medical aspects, such as organ toxicity, biomedical analysis in samples of veterinary and human fluids for metabolism screening and pharmacokinetic investigations, otherwise antibody formation after application of vaccines [29–37]. A very widely applicable HPLC-MS method has not been proposed yet [32]. Studies were directed towards specific applications and matrices, such as metabolite identification [38–44]. Therein, limits of detection (LOD) and quantitation (LOQ) were often achieved in the low microgram and nanogram per liter range [35, 45–49].

Analytical techniques of prior studies comprised fluorescence spectroscopy [45], HPLC-fluorescence detection (FD) [44], HPLC with ultra-violet (UV) detection [39, 42, 45, 47], high-performance thin layer liquid chromatography (HPTLC) [39], photodiode array (PDA) detection [40, 48], electrochemical sensoring and MnO_2_-rGO [46], ultra (U-)HPLC-diode array detection (DAD) [35, 43, 44], multi reaction monitoring (MRM) [43, 49], triple quadrupole MS [37], selected reaction monitoring (SRM) [41]. Investigated matrices consisted of distilled water, river and sewage water, plasma and saliva. Based on the findings of previous studies, concentrations of the substances and their metabolites in the aquatic environment may be expected in the pg·L^-1^ to μg·L^-1^ range [12, 27].

Hence, an analytical method was developed to detect and analyze trace substances in the concentration range described above. To this purpose, solid phase extraction, high-performance liquid chromatography interfaced by electrospray ionization (ESI), and high-resolution (HR) quadrupole time-of-flight (Q-TOF) mass spectrometry (SPE-HPLC-ESI-Q-TOF-HRMS) were employed. Chromatographic and mass spectrometric parameters were optimized. The method was applied to monitor pH-dependent stability and ozonation of FAV. Matrix effects and the method applicability was tested for a wastewater sample with added virustatic agents. The suitability for the observation of metabolites was verified for transformation products (TPs) resulting from ozonation.

## Materials and methods

### Chemicals and reagents

Antiviral drugs (name; abbreviation; % purity) were used as received. 6-fluoro-3-hydroxypyrazine-2-carboxamide (favipiravir, T-705; FAV; > 98%) was obtained from Hölzel Diagnostika Handels GmbH (Cologne, Germany). (2*S*)-2-{(2*R*,3*S*,4*R*,5*R*)-[5-(4-aminopyrrolo[2,1-*f*][1,2,4]triazine-7-yl)-5-cyano-3,4-dihydroxy-tetrahydro-furan-2-ylmethoxy]phenoxy-(*S*)-phosphorylamino}propionic acid 2-ethyl-butyl ester (remdesivir, GS-5734; REM; ≥ 99%) was purchased from BIOMOL GmbH (Hamburg, Germany). (2R,3R,4S,5R)-2-(4-aminopyrrolo[2,1-f][1,2,4]triazine-7-yl)-3,4-dihydroxy-5-(hydroxymethyl)oxolane-2-carbonitrile (GS-441524 as triphosphate, GS; ≥ 95%), ((2*R*,3*S*,4*R*,5*R*)-3,4-dihydroxy-5-(4-(hydroxyimino)-2-oxo-3,4-dihydropyrimidine-1(2*H*)-yl)tetrahydrofuran-2-yl)methyl isobutyrate (molnupiravir, EIDD-2801; MOL; 100%) and N4-hydroxycytidine (EIDD-1931; EIDD; 99.14%) were bought from Cymit Química S.L. (Barcelona, Spain). Ultrapure Berrytec water (Berrytec GmbH, Grünwald, Germany) and methanol (MeOH, ≥ 99.8%, per analysis; Bernd Kraft, Duisburg, Germany) were used to dissolve the virustatics.

### Equipment and methods

#### Analysis using HPLC-HRMS

An Agilent 1200 HPLC system (Agilent Technologies, Waldbronn, Germany) was used for chromatography. The column temperature was set to 40 °C. Reversed-phase chromatographic columns were obtained from Agilent Technologies in Waldbronn, Germany: ZORBAX Eclipse Plus C18, 150 x 2.1 mm, 3.5 µm, 95 Å; Pursuit 3 diphenyl, 50 x 2.0 mm, 3 µm, 200 Å and Polaris3Amide C18, 150 x 2.0 mm, 3 µm, 180 Å. A Kinetex core-shell silica pentafluorophenyl (PFP) + TMS endcapping (ec), 100 x 2.1 mm, 2.6 µm, 100 Å, was acquired from Phenomenex (Aschaffenburg, Germany). A hydrophilic interaction liquid chromatography (HILIC) column Nucleoshell, 150 x 3 mm, 2.7 µm, 90 Å, was bought from Macherey-Nagel (Düren, Germany) for investigations of EIDD. For an initial search of a suitable HPLC column, resolution and retention were chosen as performance criteria. Ultrapure water containing 0.1% formic acid (FA, 98-100%; Emsure; Merck KGaA, Darmstadt, Germany) was used as eluent A for HPLC. Eluent B consisted of acetonitrile (ACN, ≥ 99.9%; Carl Roth, Karlsruhe, Germany) and 0.1% FA.

The optimized eluent gradient included the following time-dependent composition: 0 min, 1% B; 1-11 min, to 99% B; 1-15 min, 99% B; 15-20 min, to 1% B. The measurement ended after 20 minutes. The flow amounted to 0.3 mL·min^-1^ during chromatographic separation, whereas the flow was raised to 0.5 mL·min^-1^ for column rinsing from minute 11 until minute 15. For flushing back to initial conditions, the flow was decreased to 0.3 mL·min^-1^ from minute 15 to 20. For further investigation of FAV, REM, MOL and GS the column ZORBAX Eclipse was eventually chosen, whereas the Nucleoshell HILIC column was selected for the analysis of EIDD and GS together with the reversed eluent gradient. Hence, the measurement started and ended with 99% ACN, but did not exceed a content of 50% water.

Following many previous studies, *e.g.* Hinnenkamp, Balsaa, Schmidt 2022 [50], an injection volume of 100 µL using full loop injection was used in order to maximize sensitivity. Recovery rate (RE) determination was carried out using 5 µL injection volume to avoid changes in peak shape due to eluate containing MeOH after SPE. The HPLC system was coupled to a Q-TOF HR-mass spectrometer (Agilent 6530 Accurate-Mass, Agilent Technologies, Waldbronn, Germany) via a Dual AJS ESI interface. Spectra were recorded in positive and negative ion mode. Ions with mass-to-charge ratio (m/z) between 50 and 1000 were detected at a scan rate of 1 spectrum/s. For MS/MS experiments, the mass range was set to 30-1000 m/z. The capillary temperature of the interface and the gas flow were adjusted to 300°C and 8 L·min^-1^. System controlling and data evaluation were carried out using MassHunter Workstation B.06.00 (Agilent Technologies, Waldbronn, Germany). Extracted ion chromatograms (EICs) were generated from total ion chromatograms by selecting the desired accurate mass. As analytical quality parameter, the retention factor *k* describing the migration rate of an analyte in the HPLC column was used.

### MS and MS/MS parameter optimization

Based on the optimized eluent gradient, s. above, the optimal MS parameters were determined: The fragmentor voltage was varied from 25 to 300 V. Skimmer voltage and nebulizer pressure were kept constant at 65 V and 14 psig. Subsequently, skimmer voltage and nebulizer pressure were varied between 30 and 75 V and 15 and 60 psig to identify the best conditions.

For FAV, REM, GS, MOL and EIDD, insufficient MS/MS mass spectra are stored in known databases, *e.g.* the National Institute of Standards and Technology (NIST) databases. In most cases, these MS/MS spectra are predicted [51–53]. Therefore, MS/MS spectra of FAV, REM, GS, MOL and EIDD were recorded. Collision-induced dissociation (CID) was used for fragmentation with nitrogen as collision gas. Collision energies (CEs/eV) were varied from 10 to 60 eV in steps of 10 eV. In order to perform multiple MS/MS experiments during one chromatographic run, targeted MS/MS was used with the precursor ions specified prior to measurement as [M+H]^+^. The mass window was set to m/z= 4.

### Method validation

The method was developed and validated according to recommendations by the German Institute of Standardization and Environmental Protection Agency [54–56]. Test parameters were LOD, LOQ, linearity, and RE as defined by the International Union of Pure and Applied Chemistry (IUPAC) [57]. Concentrations of LOD, LOQ and test for linearity of the calibration function were achieved using standard procedures [58]. The corresponding F-test was carried out by comparing the ratio of the variances of a linear and a squared function with a table value for 5% uncertainty. With respect to using signal-to-noise ratios, LOD and LOQ were determined by signal-to-noise ratio (SNR) calculation of the lowest working range concentration and extrapolation to the conditions SNR=3:1 for LOD and SNR=10:1 for LOQ.

Furthermore, RE (%) after SPE were determined by by HPLC-HRMS with the expected target concentration. Interday and intraday variations were calculated and reported as the relative standard deviation (RSD).

#### SPE, RE and wastewater matrix sample

The following cartridges were used, the maximum sorbent mass and reservoir volume as noted are given in brackets: Waters Oasis HLB3cc (60 mg, 3 mL, Waters GmbH, Eschborn, Germany), Isolute ENV+ (200 mg, 3 mL, Internationale Chemie-Technik GmbH, Bad Homburg, Germany), Chromabond Easy (200 mg, 6 mL), Chromabond C18 (ec, 500 mg, 3 mL) and Chromabond Drug (200 mg, 3 mL). Chromabond cartridges were purchased from Macherey-Nagel GmbH & Co. KG (Düren, Germany). Cartridges were washed and conditioned with 3 mL MeOH and 3 mL ultrapure water. Subsequently, the reference solutions were concentrated on the cartridge and finally eluted with 1 mL MeOH. The procedure for conditioning and equilibration was identical for the other SPE cartridges and followed the manufacturers’ instruction manuals. The capacity of both 60 mg and 200 mg maximum sorbent mass cartridges sufficed to exclude overloading. For SPE cartridge selection, Oasis HLB, Isolute ENV+, Chromabond Easy and Chromabond C18 were assayed at compound concentrations of 100 µg·L^-1^ where solutions of the compounds (20 mL) in distilled water were applied. The best performing cartridges Oasis HLB and Isolute ENV+ were subsequently tested for the five antiviral compounds at 20, 200, 500 ng·L^-1^, and 1, 2, 10 µg·L^-1^ using a sample volume of 500 mL. The solutions obtained after elution were measured by HPLC-HRMS as triple injection.

For investigation of matrix effects, a filtration effluent sample was obtained from a local WWTP (Entsorgungsgesellschaft Krefeld GmbH & Co. KG, EGK, Krefeld, Germany). The pH value was 8.2. Since the wastewater sample was found absent of the antiviral agents, the compounds were added prior to SPE. An aliquot of the sample (500 mL) was spiked with FAV, REM, GS, MOL and EIDD such that the final concentration amounted to 100 µg·L^-1^ each. The sample was concentrated using Oasis HLB and Isolute SPE cartridges described above. The SPE experiments with different wastewater sample volumes, *i.e.* 20 mL and 500 mL, and the experiments with reference solutions in distilled water or spiked sewage water were performed on different days, whereas the repeated determinations were performed on the same day. Similarly, all five analytes for the respective experimental setup were examined on the same day. The eluates of each cartridge, sample and reference solutions were measured by HPLC-HRMS in triplicate. For determining the RE in distilled water and sewage water, HPLC-HRMS measurements as triplicates of the SPE eluates were compared quantitatively with standard solutions of known concentrations. After HPLC-HRMS measurements, the samples of each cartridge, analyte and distilled water or sewage water were additionally measured by MS/MS for verification. Waters Oasis HLB3cc cartridges were selected for further study of antiviral drugs, especially for the determination of LOD and LOQ, whereas Oasis HLB and Isolute ENV+ cartridges were used for the investigation of sewage samples.

#### Method calibration

Stock solutions of each antiviral drug contained 1 mg·L^-1^ of the corresponding substance and 10% MeOH. For the calibration function ten equidistant reference points were chosen, *i.e*. for REM, GS, MOL and EIDD from 1 to 10 µg·L^-1^, and for FAV from 10 to 100 µg·L^-1^, since FAV could be evaluated with higher accuracy in the higher concentration range during range-finding tests. The samples were analyzed as triplicates and the data were tested for variance homogeneity [54–56]. Samples were processed with alternating low and high concentrations to allow for detection of carryover.

### Method application

#### Investigation of pH stability of the antiviral drugs

The investigation of pH-dependent stability of the virustatic drugs was demonstrated as application example following method optimization. For pH-dependent stability testing, 1mg·L^-1^ stock solutions of each virustatic in Berrytec water were exposed to FA and ammonia at pH values of 2.8 and 9.7. Since FAV, REM and MOL are prodrugs and their active ingredients are metabolized forms, the pH value of 2.8 was chosen based on the pH value of gastric acid. As WWTPs often operate around pH 6 to 8, compound solutions with pH 9.7, thus slightly higher, were prepared to observe possible decomposition during stability testing. The hydrolysis products were measured using the optimized HPLC-MS method with ZORBAX Eclipse column and optimized HPLC gradient.

#### Ozonation of FAV

**Fig. 1:**
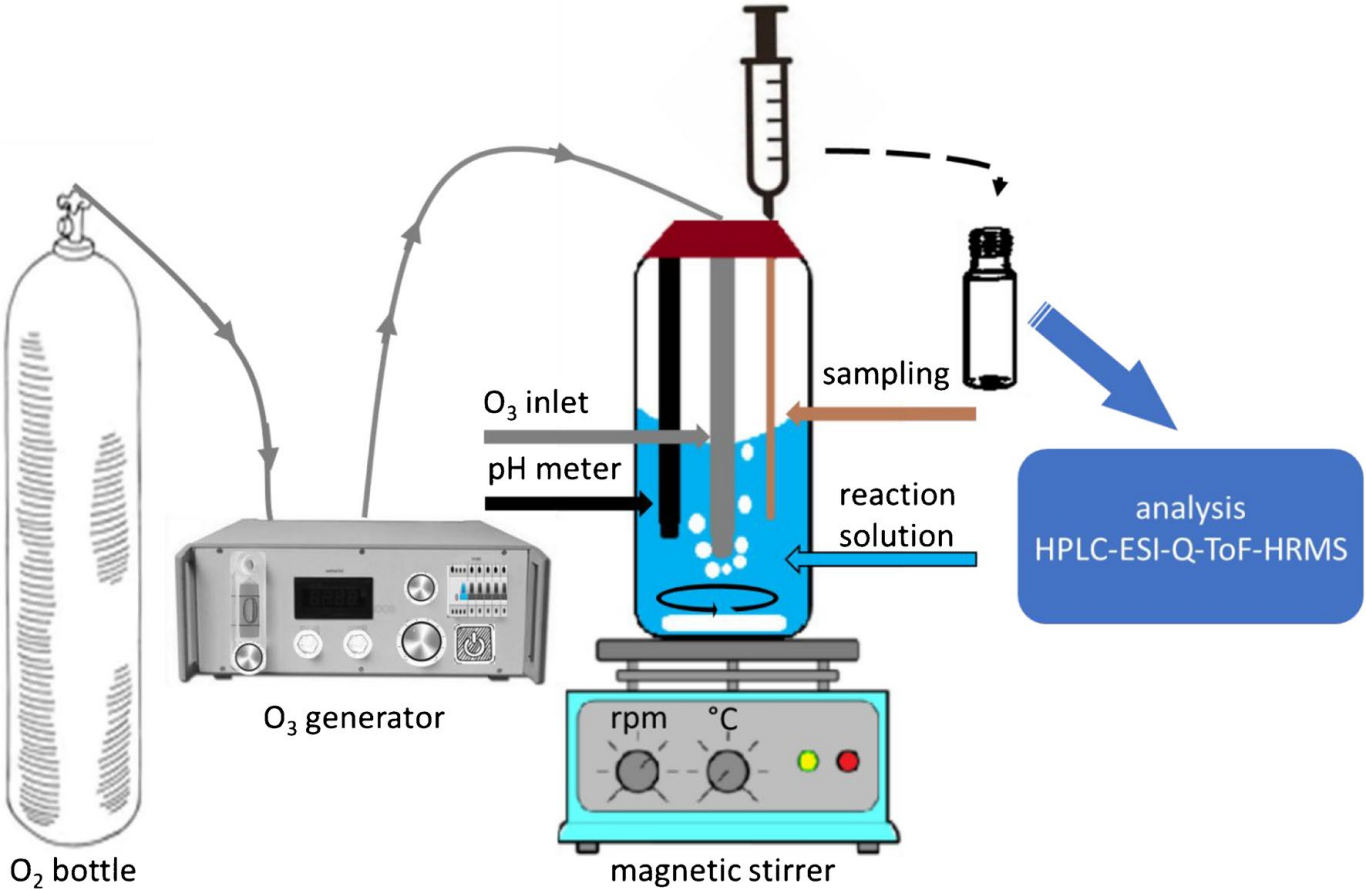
Set-up of the ozone experiment with the 0.5-L reaction vessel, O_2_ gas bottle, ozone generator, ozone inlet, pH meter, sampling and the subsequent HPLC-ESI-Q-TOF-HRMS analysis

As further application example, dissolved FAV was exposed to ozone. A 0.5-L glass vessel containing 0.5 L of the reaction solution was equipped with the gas inlet for ozone. 20.0 mg·L^-1^ of FAV were dissolved in ultrapure water containing 10% MeOH. The ozone gas was introduced from an ozone generator COM-AD-02 at 6.8 g O_3_·m^-3^ (Anseros, Klaus Nonnenmacher GmbH Tübingen, Germany), *cf*. Fig. 1.

The ozone flow through the solution was set to a rate of 25 L·h^-1^ for 30 min. The ozone content was regulated to 2.8%. Every minute, a sample of 1 mL was taken from the reaction solution. The collected samples were purged with nitrogen gas to prevent further reactions with ozone. The initial pH value of 4.5 dropped to 3.7 during ozonation. The solution temperature was kept at 19.8°C. Sample analysis was performed by HPLC-ESI-Q-TOF-HRMS without SPE. For degradation reaction monitoring, mass peak areas were plotted against ozonation time. Mass area-time curves were evaluated in normalized dimensions and were described using Matlab, version 2016b from MathWorks Inc, and pseudo first-order chemical kinetic models.

## Results and discussion

### Antiviral drugs

The chemical structures and exact masses of FAV, REM, GS, MOL, and EIDD are collected in Table 1. The accurate masses of the positive and negative quasi-molecular ions as detected by HRMS are listed together with the corresponding mass accuracy (∆*m/z*) as well. As a ∆m/z of ± 0.003 u is expected for the Q-TOF-HRMS instrument used in this study, all analytes except REM were detected in positive mode with acceptable variations. Surface activity, molecular surface and basicity influence the ionization efficiency. During the dynamic process of ionization, equilibria, kinetic effects and displacements can change the ionization efficiency [59, 60]. Hence, FAV, REM and MOL do not favor negative mode detection as can be seen from the low signal intensity and the low precision. Only EIDD and GS could be detected in negative ion mode albeit with inferior performance.

**Table 1:**
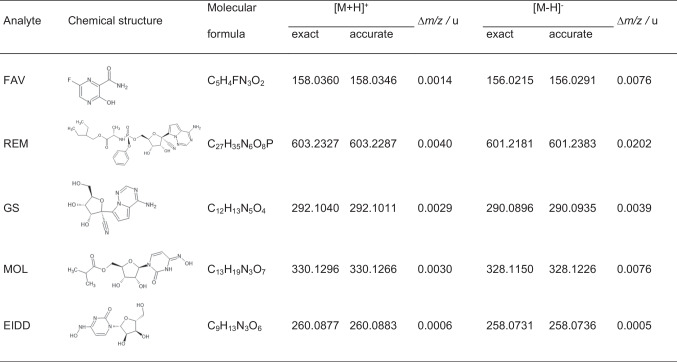
FAV, REM, GS, MOL and EIDD with their chemical structures, molecular formulas, exact and accurate mass and their absolute mass accuracies (*∆m/z*)

### HPLC parameter optimization

The ZORBAX Eclipse and Polaris3Amide columns showed good performance for the investigation of FAV, REM, GS and MOL. Resolution and retention were chosen as criteria, *cf*. supplementary information (SI) Table A1. The retention times (*R*_*t*_) and *k* values were determined for ZORBAX Eclipse. The *k* values, that were considered sufficient, are marked in italics: 5.40 min and *4.02* for FAV, 8.93 min and *7.31* for REM, 5.21 min and *3.85* for GS, 5.98 min and *4.56* for MOL and 2.41 min and 1.24 for EIDD. The Polaris3Amide column was found to be similarly well suitable for FAV, REM, GS and MOL with *R*_*t*_= 5.01, 8.70, 4.93, and 5.96°min, but insufficient for EIDD with *R*_*t*_= 1.96 min. In addition, using the Kinetex PFP column resulted in acceptable *R*_*t*_ for REM, GS and MOL, *i.e*. 7.90, 4.11 and 5.13 min, while FAV and EIDD eluted early at ≤3.23 min. The Pursuit XRs 3 diphenyl phase was suitable for analyzing REM and MOL at *R*_*t*_= 6.97 and 4.38 min, whereas FAV, GS and EIDD eluted at ≤1.40 min. The Nucleoshell HILIC column was found superior for the analysis of EIDD and GS containing samples when using the reversed eluent gradient. On this column, *R*_*t*_= 7.53 min and *k*= *2.47* were obtained for EIDD, *R*_*t*_= 7.12 min and *k*= *2.28* for GS, whereas coelution was observed for FAV, REM and MOL at retention times ≤2.79 min.

The metabolites and TPs of FAV, REM and MOL that may occur after pharmaceutical application, during wastewater treatment or in the aquatic environment, are expected to be more polar and will elute earlier on reversed phase columns. A longer *R*_*t*_ is hence preferable for the initial compound. This assumption is supported by two examples leading to transformation products: pH stability assay and ozonation of FAV, *cf*. below.

**Fig. 2:**
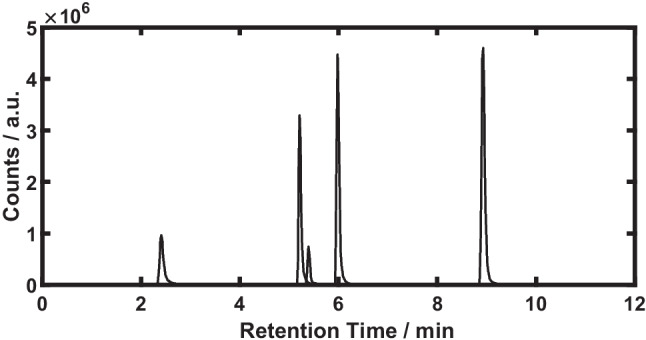
Overlay of EICs showing the measurement of EIDD (*R*_*t*_= 2.41 min), GS (*R*_*t*_= 5.21 min), FAV (*R*_*t*_= 5.40 min), MOL (*R*_*t*_= 5.98 min), and REM (*R*_*t*_= 8.93 min) using the column ZORBAX Eclipse Plus and ESI+ mode for mass detection

In summary, the five antiviral drugs were most promisingly investigated further on the column ZORBAX Eclipse using ESI+ for mass detection. An illustrative chromatogram of the five analytes using the ZORBAX Eclipse column, which provided good resolution with a good SNR, is shown in Fig. 2.

### MS and MS/MS parameter optimization

For best results with respect to peak intensity, fragmentor voltages of 125 V for FAV, 200 V for REM and GS, 150 V for MOL and 100 V for EIDD were applied. For EIDD, a lower voltage had to be chosen as in-source fragmentation occurred at higher voltages. The pentose moiety was cleaved as will be discussed below. Skimmer voltage optimization yielded 55 V for FAV and EIDD and 70 V for REM, GS and MOL. Yet, voltages between 55 and 75 V did not affect the signal intensities of REM, GS and MOL strongly. A nebulizer pressure of 30 psig proved best for FAV, REM, GS and MOL, whereas 20 psig was suitable for EIDD.

**Fig. 3:**
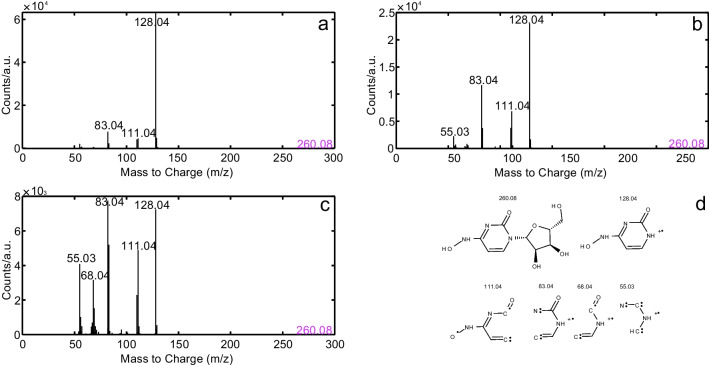
MS/MS spectra of EIDD at CE= 20 eV (**a**), 30 eV (**b**) and 40 eV (**c**) and fragment identification (**d**)

Experimental MS/MS parameter optimization for Q-TOF instruments will not directly enhance the sensitivity of the method, but affects the number of fragments obtained. The number of specific fragments increases the identification certainty and indirectly the sensitivity as the number of ions is distributed over the number of fragments. The detected MS/MS fragments are described below. The MS/MS spectra providing the most significant difference are displayed. For FAV, 10 eV were sufficient to yield four characteristic fragments. Higher CEs, *e.g.* 60 eV, led to only one fragment, *i.e.* [M+H]^+^= 58.01, *cf*. SI Figure A1. For REM, collision energies as low as 10 and 20 eV caused highly specific fragmentation, whereas CEs of 30 eV and above gave rise to a single remaining fragment, [M+H]^+^= 200.04, which proved stable up to 60 eV [44], *cf*. SI Figure A2. For GS, 10 eV were not sufficient to form an observable fragment at all. Without significant differences, the most meaningful fragments were detected at 30 and 40 eV, *cf*. SI Figure A3. Only three fragments were found for MOL at CE= 60 eV. At 10 eV, a single fragment [M+H]^+^= 128.04 was obtained, *cf*. SI Figure A4. EIDD’s fragments after MS/MS-experiments are given in Fig. 3.

In this study, the main fragment [M+H]^+^= 128.04 was identified through MS/MS experiments as N4-hydroxycytosine [61]. Its formation originates from the cleavage of the pentose moiety. With increasing CEs, N4-hydroxycytosine fragments having m/z values of 83.04, 111.04, 55.03 and 68.04 occurred. In summary, low collision energies of 10 to 20 eV were found to be optimal for FAV and REM, medium to high CEs of 30-40 eV for GS and EIDD and the highest CE of 60 eV for MOL.

### Method validation: SPE, RE and wastewater sample

For sample concentration and matrix removal, SPE may precede HPLC-HRMS analysis. Elution condition variation for Oasis HLB, Isolute ENV+, Chromabond Easy, Chromabond Drug, and Chromabond C18 showed that ACN led to insufficient elution. Despite of its lower elution strength on reversed-phases, MeOH proved more selective and hence suitable for the compounds under investigation. REs served as quality criterion. The tested SPE cartridges Chromabond Drug, Chromabond Easy, and Isolute ENV+ did not provide sufficiently high REs for all compounds. Exemplarily, Isolute ENV+ yielded 26, 3, 7, 36 and 33% for FAV, REM, GS, MOL and EIDD, respectively. Good REs were found using Oasis HLB cartridges for FAV, REM, GS and MOL amounting to 61, 109, 106, 104%, and Isolute ENV+ for EIDD amounting to 33%, *cf*. Table 2. FAV was not equally retained by the non-polar C18 phase. When reducing the sample concentrations from 100 µg·L^-1^ to 20 ng·L^-1^, REM, GS and MOL were successfully detected using Oasis HLB.

**Table 2:** Average REs (%) and RSD (%) for SPE of FAV, REM, GS, MOL and EIDD using Oasis HLB, Isolute ENV+, Chromabond Easy and Chromabond C18 cartridges indicating the sample matrix, the sample volume and the determined concentration

cartridge	matrix	sample volume / mL	c / µg·L^-1^	RE / %; RSD / %				
				FAV	REM	GS	MOL	EIDD
HLB	dist. water	20	1·10^2^	61; 4	109; 5	106; 4	104; 5	3; <1
ENV+	dist. water	20	1·10^2^	26; 1	3; <1	7; <1	36; 1	33; 1
Easy	dist. water	20	1·10^2^	23; 5	71; 8	71; 9	65; 4	14; 2
C18	dist. water	20	1·10^2^	n.a.	113; 4	123; 3	94; 9	12; 2
HLB	dist. water	500	2·10^-2^	n.a.	115; 1	64; 15	100; 2	n.a.
HLB	dist. water	500	2·10^-1^	n.a.	80; 1	86; 1	82; 1	n.a.
HLB	dist. water	500	5·10^-1^	n.a.	80; 1	85; 1	82; 1	n.a.
HLB	dist. water	500	1	n.a.	80; 1	83; 3	85; 1	n.a.
HLB	dist. water	500	2	n.a.	90; 1	77; 1	92; 1	n.a.
HLB	dist. water	500	1·10^1^	8; 1	99; 3	94; 2	91; 3	2; <1
ENV+	dist. water	500	2·10^-1^	n.a.	8; <1	23; <1	47; 1	n.a.
ENV+	dist. water	500	5·10^-1^	n.a.	1; <1	2; <1	9; <1	n.a.
ENV+	dist. water	500	1	n.a.	<1; <1	n.a.	2; <1	7; <1
ENV+	dist. water	500	2	42; 3	<1; <1	n.a.	2; <1	7; <1
ENV+	dist. water	500	1·10^1^	27; 1	<1; <1	n.a.	4; <1	22; <1
HLB	sewage	500	1·10^1^	n.a.	62; 2	59; 1	58; 1	2; <1
ENV+	sewage	500	1·10^1^	n.a.	20; <1	33; <1	27; <1	32; 1

Yet, only 8% of FAV and 2% of EIDD were recovered at their highest concentration, *i.e*. 10 µg·L^-1^, from a sample of 500 mL distilled water using Oasis HLB. In contrast, using the Isolute ENV+ cartridge, 42% of FAV could be recovered at 2 µg·L^-1^ and 27% at 10 µg·L^-1^. For EIDD, 7% were recovered at 1 and 2 µg·L^-1^ and 22% at 10 µg·L^-1^. The Oasis HLB cartridge has been found to have a good RE in the concentration range between 20 ng·L^-1^ and 10 µg·L^-1^ for REM, GS and MOL in distilled water ranging from 64 to 115%.

During the investigation of the spiked wastewater sample, FAV at 10 µg·L^-1^ was not recovered using Oasis HLB and Isolute ENV+. For EIDD, the Isolute ENV+ cartridge showed a RE of 32%, which is somewhat higher than that from distilled water. The Oasis HLB cartridge yielded REs between 58 and 62% for REM, GS and MOL, which was inferior to those from distilled water. The Isolute ENV+ cartridge proved also suitable, albeit of poorer performance as compared to the Oasis HLB, for REM, GS and MOL with REs between 20 and 33%, while no acceptable REs were observed with distilled water.

Intraday precision was tested for the Oasis HLB cartridge with 20 mL sample volume of distilled water and concentrations of 100 µg·L^-1^. The precision was found acceptable with RSDs of 4, 5, 4, 5 and <1% for FAV, REM, GS, MOL, and EIDD, *cf*. Table 2. Only GS showed intraday precision of 15% during investigation of Oasis HLB and 500 mL sample volume. The interday precision was determined using the Oasis HLB cartridge and 500 mL sample volume of distilled water for FAV, REM, GS, MOL, and EIDD to 1, 12, 10, 6 and <1%. Interday precision for FAV, REM, GS, MOL and EIDD using the Isolute ENV+ cartridge resulted to 8, 4, 11, 18 and <1%, respectively. In total, the Oasis HLB cartridge showed the best overall performance as it has often been reported for aquatic environmental analysis of other micropollutants. Only for EIDD, the more polar Isolute ENV+ yielded better REs. Concentrations of the antiviral agents were varied to test for linearity of RE. Oasis HLB and Isolute ENV+ cartridges: From Table 2, it can be seen that FAV and EIDD did not yield reasonable RE with Oasis HLB and Isolute ENV+ cartridges, nor did REM, GS, and MOL on the latter cartridge. After testing and exclusion of the outliers 20 ng·L^-1^ REM, GS and MOL and also of 2 µg·L^-1^ GS and MOL, the values indicated first constant, then increasing RE with increasing concentrations. The outliers at the lowest concentrations showed the highest RE for REM and MOL. Although linearity was not confirmed over the range from 20 ng·L^-1^ to 10 µg·L^-1^, Oasis HLB was hence found suitable for separation and isolation of REM, GS, and MOL from a distilled water matrix.

### Method validation: LOD and LOQ

Values collected from previous studies were based on different ways to determine LOD and LOQ, *i.e.* by calibration function, SNR and SPE, and should hence be compared with caution. The values for the five antiviral compounds together with analytical method, application fields, and calibration function or SNR approach are listed in Table 3. In this study, a linear calibration function was observed and verified against a squared function for all five analytes in their working range of 1 to 10 µg·L^-1^ or 10 to 100 µg·L^-1^. The use of HPLC-HRMS for FAV analysis in this study led to superior sensitivity as compared to previously reported HPLC-UV and HPLC-fluorescence methods [45]. Yet, the best overall performance was reported when using SPE-HPLC-MS/MS [62]. In the current study, the use of SPE yielded additional improvement in LOD and LOQ values. For FAV, similarly good LODs and LOQs were not attained due to the low REs. As expected, MS detection proved superior to absorption detection. The best LOD and LOQ with the method described here were obtained for REM. As to MS techniques, the application of MRM did not prove superior to HPLC-HRMS [43, 49]. Analogously for GS, lower LOD and LOQ were determined using HPLC-HRMS without SPE than using UHPLC-triple quadrupole MS [37]. LOD and LOQ for MOL and EIDD without SPE as determiend in this study were comparable to values from previous reports [41], but also profited from the use of SPE. It was concluded from overall comparison that the determination of LOD and LOQ by SNR yielded very comparable values to that by calibration function, where differenced did not exceed a factor of 5. While MRM is often associated with the highest sensitivity, comparable LOD and LOQ values were obtained in this study using HPLC-HRMS [43]. The combination of SPE and HPLC-HRMS resulted in lower, *i.e.* better, LOD and LOQ than had been reported before, thus rendering the method suitable for environmental analysis both with respect to selectivity and sensitivity. In addition, the treatment of the wastewater sample, albeit spiked, proved that detection and quantitation of REM, GS and MOL using the Oasis HLB cartridge was possible.

**Table 3:** LOD and LOQ of FAV, REM, GS, MOL and EIDD using the analytical method and determined through calibration function, SNR and including SPE prior to analytical method

Compound	Analytical method	Approach for LOD and LOQ determination	Matrix	LOD / µg·L^-1^	LOQ / µg·L^-1^	Literature
FAV	HPLC-HRMS	calibration function	ultrapure water	2.8	9.2	This study
	HPLC-HRMS	SNR 3:1, 10:1	ultrapure water	1.3·10^1^	4.2·10^1^	This study
	SPE-HPLC-HRMS, HLB	SNR 3:1, 10:1	ultrapure water	2.1·10^-1^	6.9·10^-1^	This study
	SPE-HPLC-HRMS	SNR 3:1, 10:1	sewage water	n.a.	n.a.	This study
	Fluorescence spectroscopy	calibration function	ultrapure water	4.0	1.1·10^1^	[45]
	HPLC-UV	calibration function	ultrapure water	9.0·10^2^	3.0·10^3^	[45]
	SPE-HPLC-MS/MS	calibration function	river and sewage water	/	4·10^-4^	[62]
	Electrochemical sensor, MnO_2_-rGO	calibration function	plasma	1.4	4.6	[46]
	UHPLC-DAD	calibration function	plasma	/	LLOQULOQ: 1·10^2^1·10^4^	[35]
	HPLC-UV	SNR 3:1, 10:1	serum and plasma	1.2·10^3^	3.6·10^3^	[47]
	HPLC-PDA	SNR 3:1, 10:1	ultrapure water	1.8·10^2^	5.3·10^2^	[48]
	HPLC-UV	SNR 3:1, 10:1	distilled water	7.2·10^1^	2.2·10^2^	[42]
REM	HPLC-HRMS	calibration function	ultrapure water	6.0·10^-1^	1.8	This study
	HPLC-HRMS	SNR 3:1, 10:1	ultrapure water	2.0·10^-1^	6.0·10^-1^	This study
	SPE-HPLC-HRMS, HLB	SNR 3:1, 10:1	ultrapure water	1.8·10^-3^	5.5·10^-3^	This study
	SPE-HPLC-HRMS, HLB	SNR 3:1, 10:1	sewage water	2.7·10^-1 b)^	8.9·10^-1 b)^	This study
	HPTLC-UV	SNR 3:1, 10:1	50% aqueous ethanol	1.7·10^3^	5.6·10^3^	[39]
	HPLC-MS/MS using MRM	calibration function	water / ACN (50/50)	7.0·10^-1^	1.3	[49]
	UPLC-MS/MS using MRM	calibration function	plasma	/	9.8·10^-1^, 1.0, 2.0, 4.0, 5.0	[43]
	UPLC-DAD	calibration function	plasma	/	5.0	[43]
	UHPLC-DAD	calibration function	plasma	/	LLOQ → ULOQ: 1·10^2^ → 1·10^4^	[35]
	UHPLC-MS/MS	calibration function	plasma	/	LLOQ → ULOQ: 4.0 → 4.0·10^3^	[37]
	HPLC-PDA	SNR 3:1, 10:1	mobile phase ^a)^	5.0·10^2^	2.0·10^3^	[40]
	HPLC-DAD	SNR 3:1, 10:1	MeOH	3.0·10^1^	1.0·10^2^	[44]
	HPLC- FD	SNR 3:1, 10:1	MeOH	1.5·10^1^	5.0·10^1^	[44]
GS	HPLC-HRMS	calibration function	ultrapure water	3.0·10^-1^	9.0·10^-1^	This study
	HPLC-HRMS	SNR 3:1, 10:1	ultrapure water	2.0·10^-1^	8.0·10^-1^	This study
	SPE-HPLC-HRMS, HLB	SNR 3:1, 10:1	ultrapure water	1.9·10^-3^	7.6·10^-3^	This study
	SPE-HPLC-HRMS, HLB	SNR 3:1, 10:1	sewage water	2.7 ^b)^	8.9 ^b)^	This study
	UPLC-MS/MS using MRM	calibration function	plasma	/	9.8·10^-1^, 2.0, 5.0	[43]
	UHPLC-MS/MS	calibration function	plasma	/	LLOQ → ULOQ: 2 → 2.0·10^3^	[37]
MOL EIDD	HPLC-HRMS	calibration function	ultrapure water	3.0·10^-1^	1.0	This study
	HPLC-HRMS	SNR 3:1, 10:1	ultrapure water	3.0·10^-1^	9.0·10^-1^	This study
	SPE-HPLC-HRMS, HLB	SNR 3:1, 10:1	ultrapure water	2.9·10^-3^	8.7·10^-3^	This study
	SPE-HPLC-HRMS, HLB	SNR 3:1, 10:1	sewage water	8.8·10^-1 b)^	2.9 ^b)^	This study
	MS/MS using SRM and ESI-	calibration function	plasma and saliva	/	2.5	[41]
	HPLC-HRMS	calibration function	ultrapure water	6.0·10^-1^	1.7	This study
	HPLC-HRMS	SNR 3:1, 10:1	ultrapure water	4.0·10^-1^	1.2	This study
	SPE-HPLC-HRMS, HLB	SNR 3:1, 10:1	ultrapure water	1.3·10^-1^	3.8·10^-1^	This study
	SPE-HPLC-HRMS, ENV+	SNR 3:1, 10:1	sewage water	3.6 ^b)^	1.2·10^1 b)^	This study
	MS/MS using SRM and ESI-	calibration function	plasma and saliva	/	2.5	[41]

^a^ 0.025 M polyoxyethylene, 0.1 M sodium lauryl sulfate and 0.02 M of disodium hydrogen phosphate in 1 L of de-ionized water [40]

^b^ Values obtained from extrapolation of the recovered concentration

Detectable and quantifiable concentrations may amount to 2.7·10^-1^ and 8.9·10^-1^ µg·L^-1^ for REM, 2.7 and 8.9 µg·L^-1^ for GS, and 8.8·10^-1^ and 2.9 µg·L^-1^ for MOL. For EIDD, LOD and LOQ were extrapolated to 3.6 and 1.2·10^1^ µg·L^-1^ when including the Isolute ENV+ cartridge.

### Investigation of the pH stability of the antiviral drugs

After method optimization, pH-dependent stability was monitored to serve as an application example. REM, GS and MOL were found stable at pH 2.8, since no by-products or TPs were observed. The mass peak area of the initial compound was observed constant. In contrast, EIDD underwent decomposition of about 14% immediately and FAV completely decomposed [63]. FAV, REM, MOL and EIDD were degraded completely at pH 9.7, whereas EIDD was instable and decreased by 49% [63]. The TPs resulted from hydrolysis as expected. No products were detected for FAV. The hydrolysis products of REM at pH 9.7 were characterized by the quasi molecular ions [M+H]^+^= 527.1906 and 443.1001 at *R*_*t*_= 8.99 and 6.54 min. The corresponding observations for GS were [M+H]^+^= 310.1100 at *R*_*t*_= 1.59, for MOL [M+H]^+^= 260.0883, 128.0491 and 244.0911 at *R*_*t*_= 2.02, 2.02 and 1.88 min and for EIDD [M+H]^+^= 128.0491, 244.0911 and 487.2236 at *R*_*t*_= 2.02, 1.93 and 1.95 min, *cf*. SI Figure A5. Alkaline media led to cleavage of the active agent EIDD with [M+H]^+^= 260.0883. The quasi molecular ions [M+H]^+^= 128.0491 and 244.0911 were decomposition products identical for MOL and EIDD, as expected. The ion [M+H]^+^= 128.0491 was identified as N4-hydroxycytosine, *cf*. above. Its occurrence was explained in terms of in-source fragmentation. The TP [M+H]^+^= 244.0911 represented a hydroxyl group elimination. The ion with m/z= 487.2236 was interpreted as the dimer [2M+H]^+^ of [M+H]^+^= 244.0911 formed during ionization in the mass spectrometer. The MS/MS spectra of the hydrolysis products are included in SI Figure A6.

### Ozonation of FAV

**Fig. 4:**
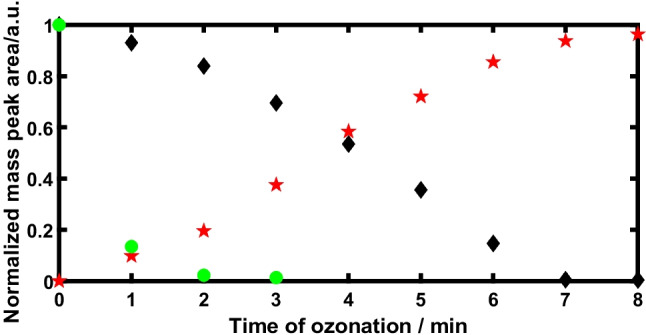
Normalized (c/c_0_) concentration-time profiles of the decomposition of 20 mg·L^-1^ of FAV (black diamonds) during ozonation over 30 minutes and the decrease of a product [M+H]^+^= 174.10 initially present (green dots) and the increase of a TP [M+H]^+^= 172.03 due to ozonation (red pentagrams)

As shown above, FAV could be identified at a level of 2.8 µg·L^-1^ using the developed HPLC-HRMS method by calibration function and without SPE. Starting from an initial concentration of 20 mg·L^-1^ FAV, the ozone-induced decomposition was monitored. A quantity of 80 µg·L^-1^ was detected after 8 min. At this point, 99.6% of FAV were hence transformed. On inspection of the mass spectra, most TPs showed a loss of the fluorine atom or its substitution by a hydroxyl group in agreement with previous findings [64, 65]. The time courses of the degradation reaction of FAV, an initial by-product ([M+H]^+^= 174.10) and a product formed on ozonation and persisting after 30 min ([M+H]^+^= 172.03) are shown in Fig. 4.

The concentration-time curves of the following TPs were determined as mass-area *vs*. time during ozone treatment and were characterized by their quasi-molecular ions [M+H]^+^, *cf*. Table 4. Five TPs were formed in the beginning of ozone treatment and were transformed or degraded during the 30 minutes of treatment. Eight TPs persisted at the end of the ozonation.

**Table 4:** TPs ([M+H]^+^) of favipiravir after ozone treatment, indicating the time of their formation and the determined retention time (*R*_*t*_) using the optimized HPLC-HRMS method

Type of product	[M+H]^+^	*R*_*t*_ / min
formed and observed at the beginning of ozonation	156.09	7.55
	170.11	8.14
	174.10	7.55
	188.12	8.14
	210.10	8.14
Persistent and observed at the end of ozonation	104.03	2.03
	160.03	3.03
	172.03	1.19
	190.04	1.35
	203.95	2.03
	204.05	2.03
	205.04	1.77
	206.04	1.77

The structural elucidation of the detected TPs is possible by analyzing the MS/MS spectra, *cf*. above, and will be discussed elsewhere

## Conclusion

For the three approved virustatic drug substances FAV, REM, MOL, and the two active metabolites GS and EIDD, an analytical method was developed that allows trace analysis in aqueous samples. Limits of detection and quantitation were achieved in the nanogram per liter range for REM, GS and MOL and in the hundreds of nanogram per liter range for FAV and EIDD. The method comprised SPE, HPLC using a gradient eluent and HRMS. An Oasis HLB proved most versatile towards a distilled water matrix, whereas the more polar EIDD profited from an Isolute ENV+ cartridge. For FAV, a higher working range was required than for the other antiviral agents. Wastewater matrix effects reduced the REs obtained with Oasis HLB for FAV, REM, GS, and MOL in distilled water, but rendered the Isolute ENV+ applicable for EIDD, REM, GS, and MOL. Using SNR LOQ and LOQ calculation, the method presented here was comparable in sensitivity to HPLC-MS/MS and MRM techniques. Including SPE led to further improvement of LOD and LOQ. On testing pH stability, the method proved suitable for the detection of TPs. Decomposition and transformation of FAV could be monitored during ozone treatment, where normalized concentration-time profiles of the initial compound and its TPs were recorded. The method may hence contribute to the trace analysis of surface water and effluents for antiviral drugs and their metabolites. On continuation of the SARS-CoV-2 pandemic and its treatment, the investigated and future drugs are expected to enter the aquatic environment, requiring sensitive analytical methods suitable for monitoring. The method may also support structure elucidation of TPs after treatment by advanced oxidation processes (AOPs) such as ozonation and ecotoxicological assays to determine potential hazard of new and unknown TPs.

## Supplementary Information

Below is the link to the electronic supplementary material.

Supplementary file1 (DOCX 3169 KB)

## Data Availability

The data presented in this study are available on request from the corresponding author. The link will be provided upon request.
