# Supplementary material for: Determination of anti-SARS-CoV-2 virustatic pharmaceuticals in the aquatic environment using high-performance liquid chromatography high-resolution mass spectrometry
Source: Anal Bioanal Chem. 2023 Jul 13;415(22):5365–77. doi: 10.1007/s00216-023-04811-7 (PMC10444687; doi:10.1007/s00216-023-04811-7)
Supplement: Supplementary file 1 — Supplementary file1 (DOCX 3169 KB) [file 216_2023_4811_MOESM1_ESM.docx]

**Analytical and Bioanalytical Chemistry**

Supplementary information

**Determination of anti-SARS-CoV-2 virustatic pharmaceuticals in the aquatic environment using high-performance liquid chromatography high-resolution mass spectrometry**

**Indra Bartels ^a,b^, Martin Jaeger ^a,*^, Torsten C. Schmidt ^b^**

^a^ Department of Chemistry and ILOC, Niederrhein University of Applied Sciences, Frankenring 20, D-47798 Krefeld, Germany, ORCID ID: 0000-0001-7245-443X; ^*^ corresponding author: martin.jaeger@hs-niederrhein.de, ORCID ID: 0000-0002-7709-2869; ^b^ Faculty of Chemistry, University Duisburg-Essen, Universitätsstraße 5, D-45141 Essen, Germany, ORCID ID: 0000-0003-1107-4403

Table A1 Retention times *R_t_* and retention factors *k* from HPLC-HRMS chromatograms (ESI+ measurement) of FAV, REM, GS, MOL and EIDD on the columns ZORBAX Eclipse Plus (1), Pursuit XRs 3 diphenyl (2), Kinetex PFP (3), Polaris3Amide (4) and Nucleoshell HILIC (5). Sufficient *k* are marked in italics

| Drug | *R_t_* / min and *k* | Column No. | | | | |
| --- | --- | --- | --- | --- | --- | --- |
|  |  | 1 | 2 | 3 | 4 | 5 |
| FAV | *R_t_* | 5.40 | 1.25 | 3.23 | 5.01 | 2.32 |
|  | *k* | *4.02* | 1.18 | *3.77* | *4.71* | 0.07 |
| REM | *R_t_* | 8.93 | 6.97 | 7.90 | 8.70 | 2.79 |
|  | *k* | *7.31* | *11.16* | *10.67* | *8.92* | 0.29 |
| GS | *R_t_* | 5.21 | 1.40 | 4.11 | 4.93 | 7.12 |
|  | *k* | *3.85* | 1.44 | *5.07* | *4.62* | *2.28* |
| MOL | *R_t_* | 5.98 | 4.38 | 5.13 | 5.96 | 2.79 |
|  | *k* | *4.56* | *6.64* | *6.58* | *5.80* | 0.29 |
| EIDD | *R_t_* | 2.41 | 0.78 | 1.18 | 1.96 | 7.53 |
|  | *k* | 1.24 | 0.36 | 0.74 | 1.23 | *2.47* |


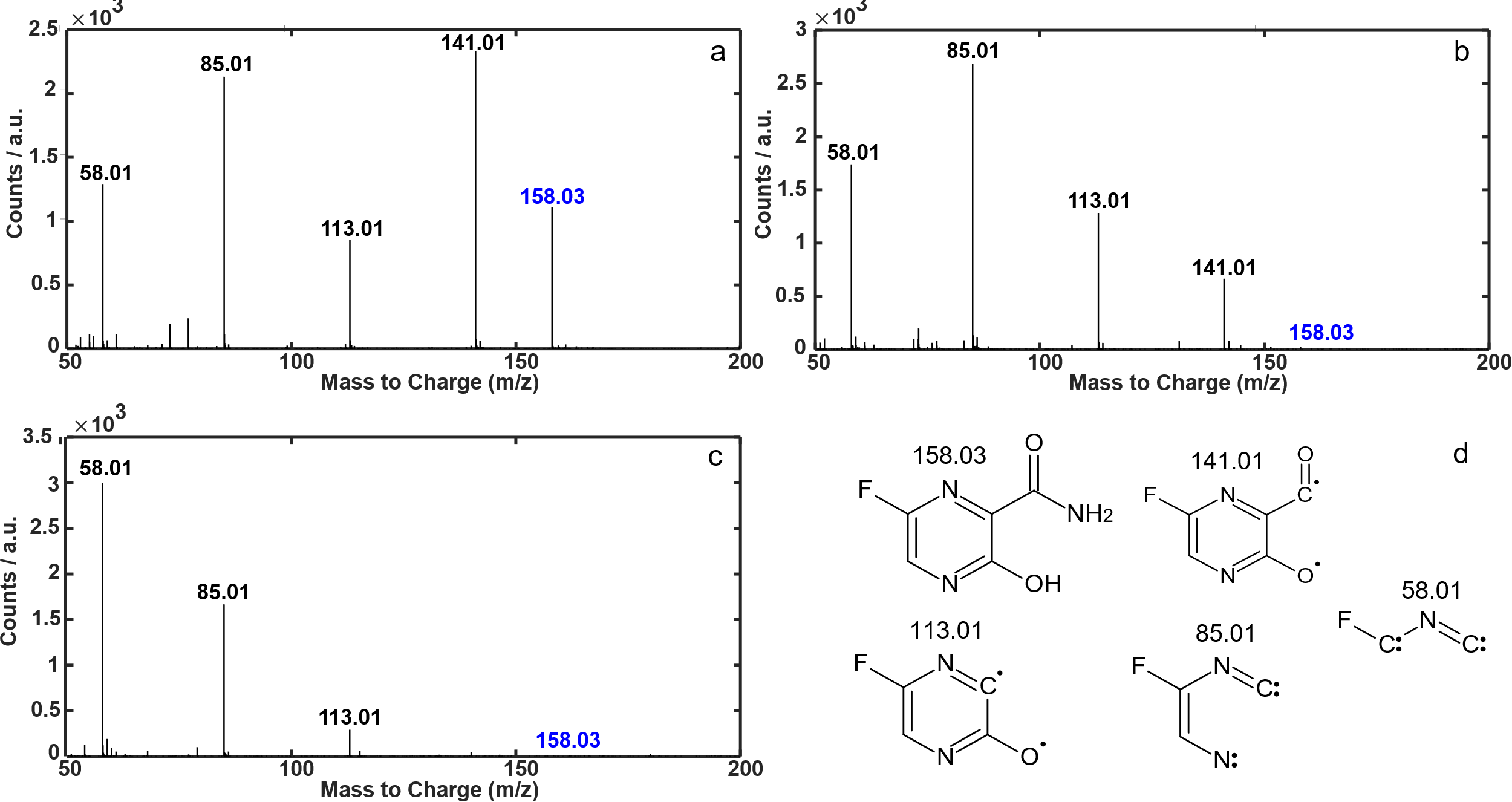


Figure A1 MS/MS spectra of FAV at CE= 10 eV (a), 20 eV (b) and 30 eV (c) and fragment identification (d)


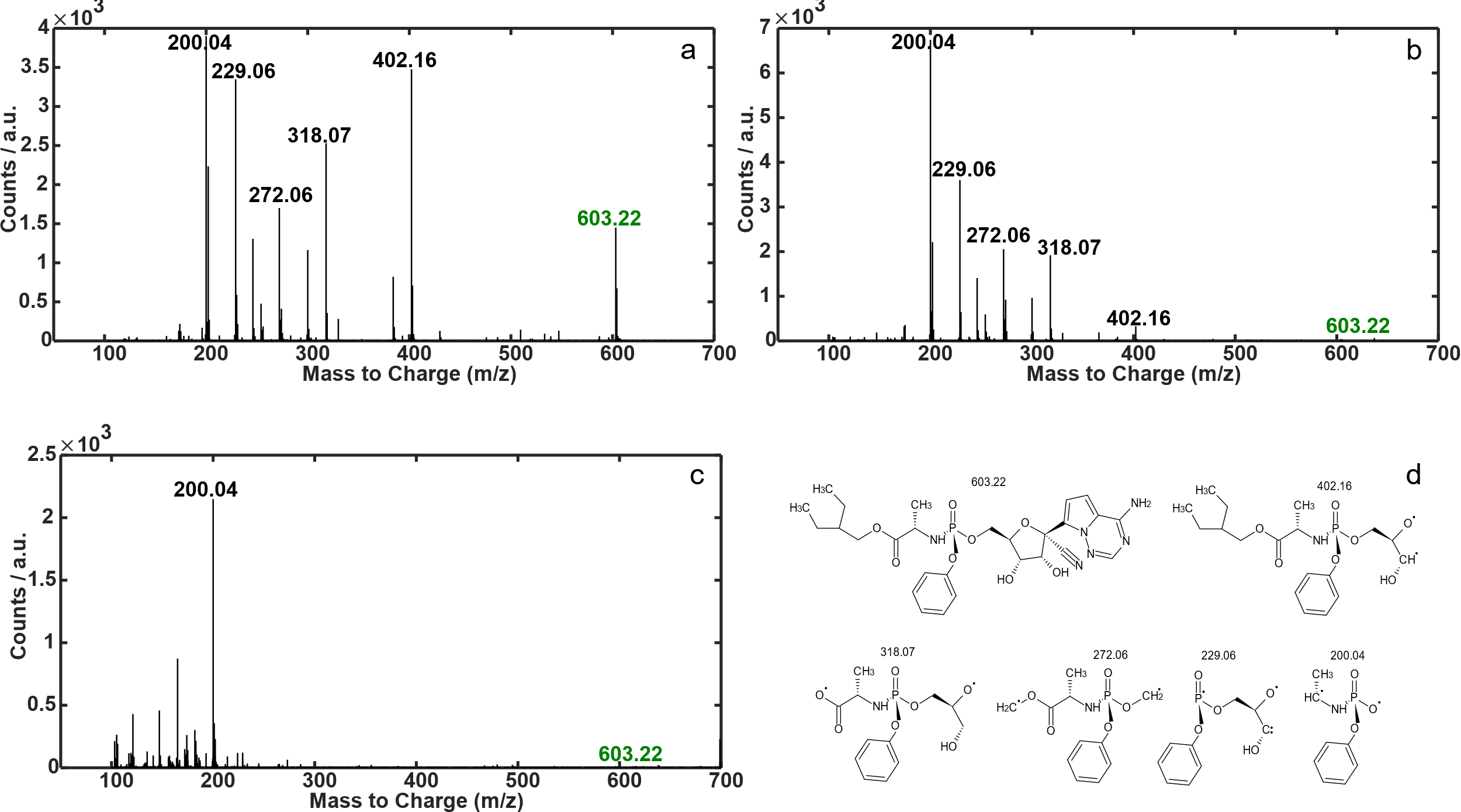


Figure A2 MS/MS spectra of REM at CE= 10 eV (a), 20 eV (b) and 60 eV (c) and fragment identification (d)


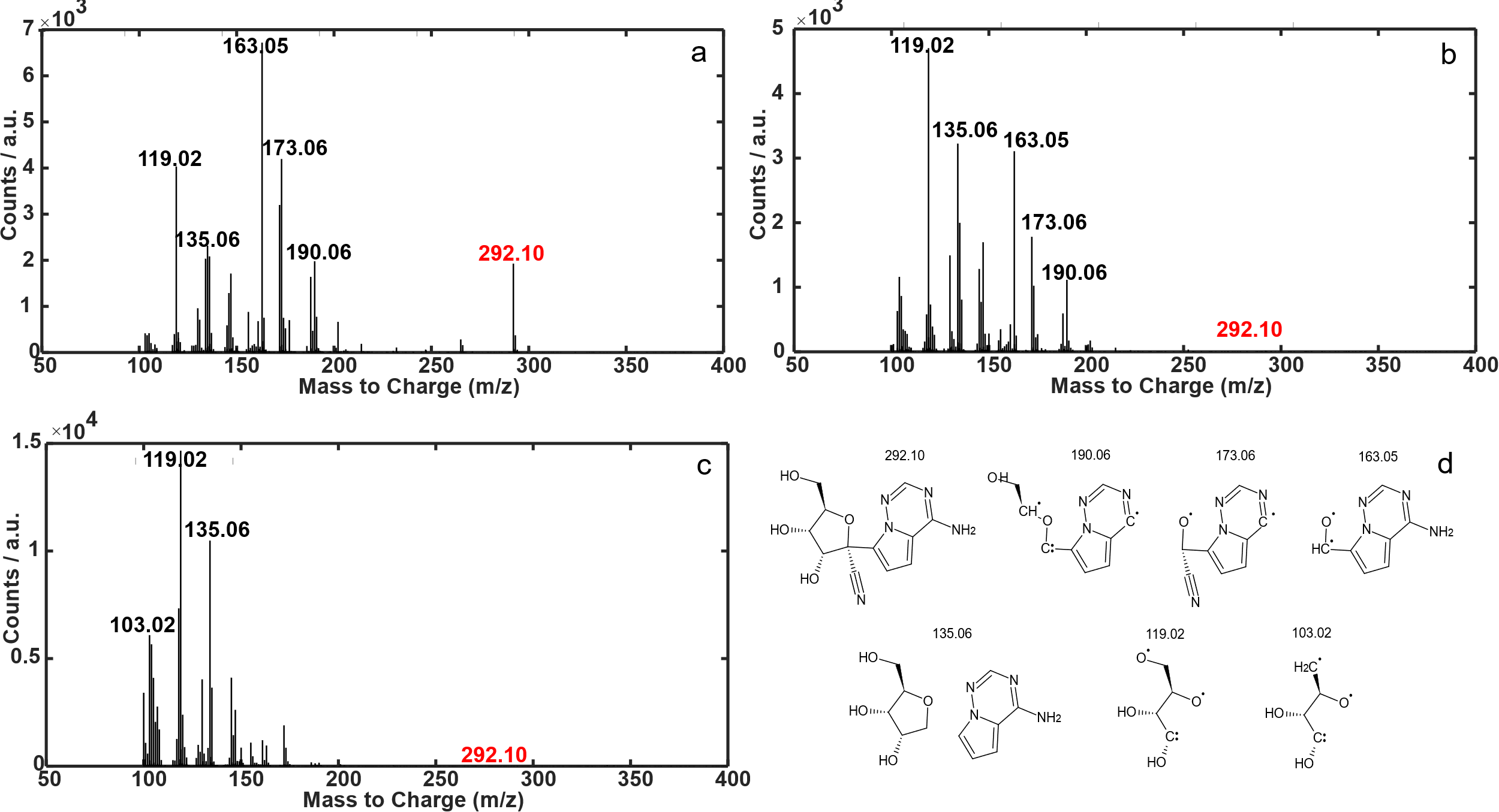


Figure A3 MS/MS spectra of GS at CE= 30 eV (a), 40 eV (b) and 60 eV (c) and fragment identification (d)


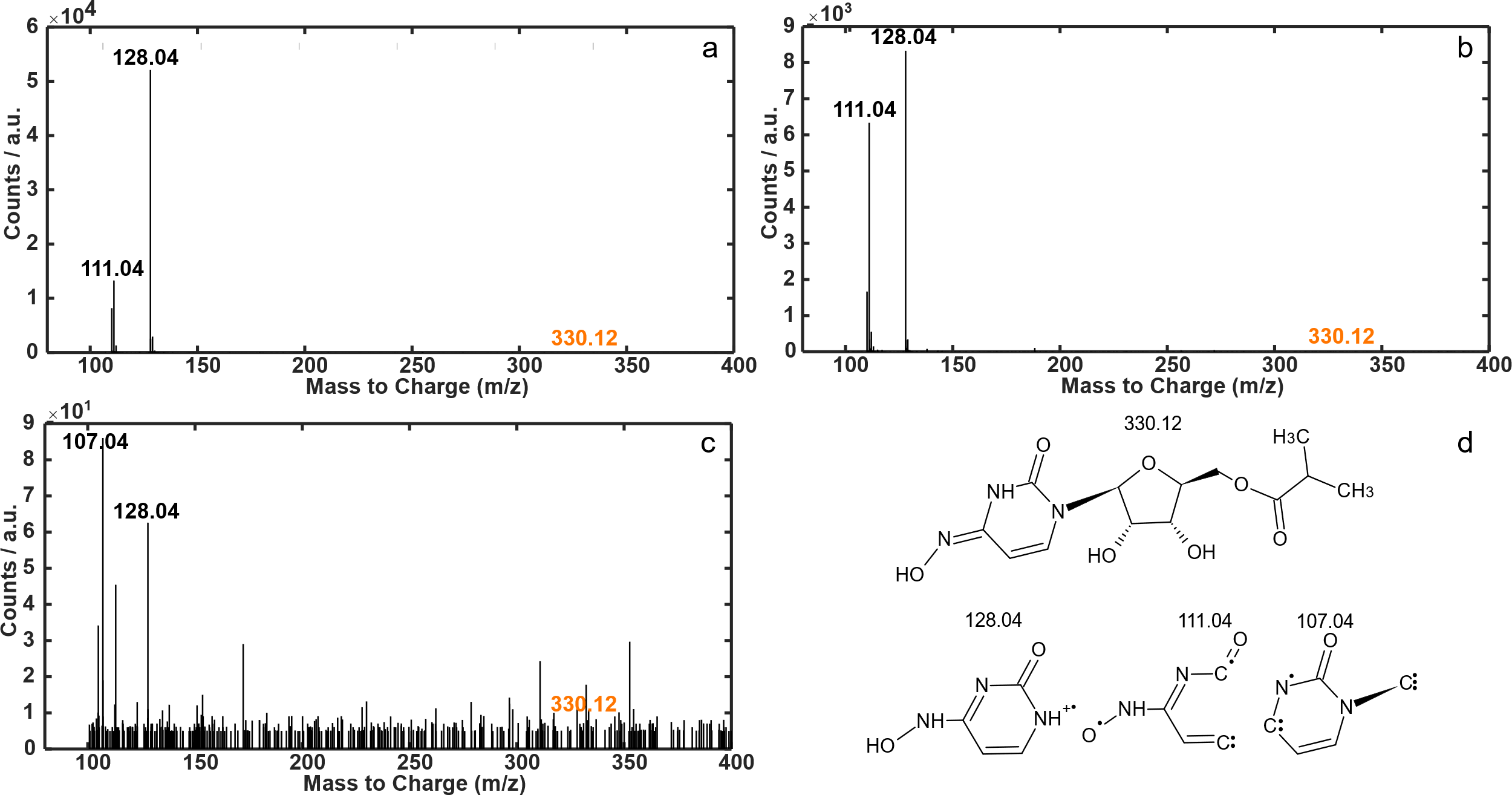


Figure A4 MS/MS spectra of MOL at CE= 40 eV (a), 50 eV (b) and 60 eV (c) and fragment identification (d)


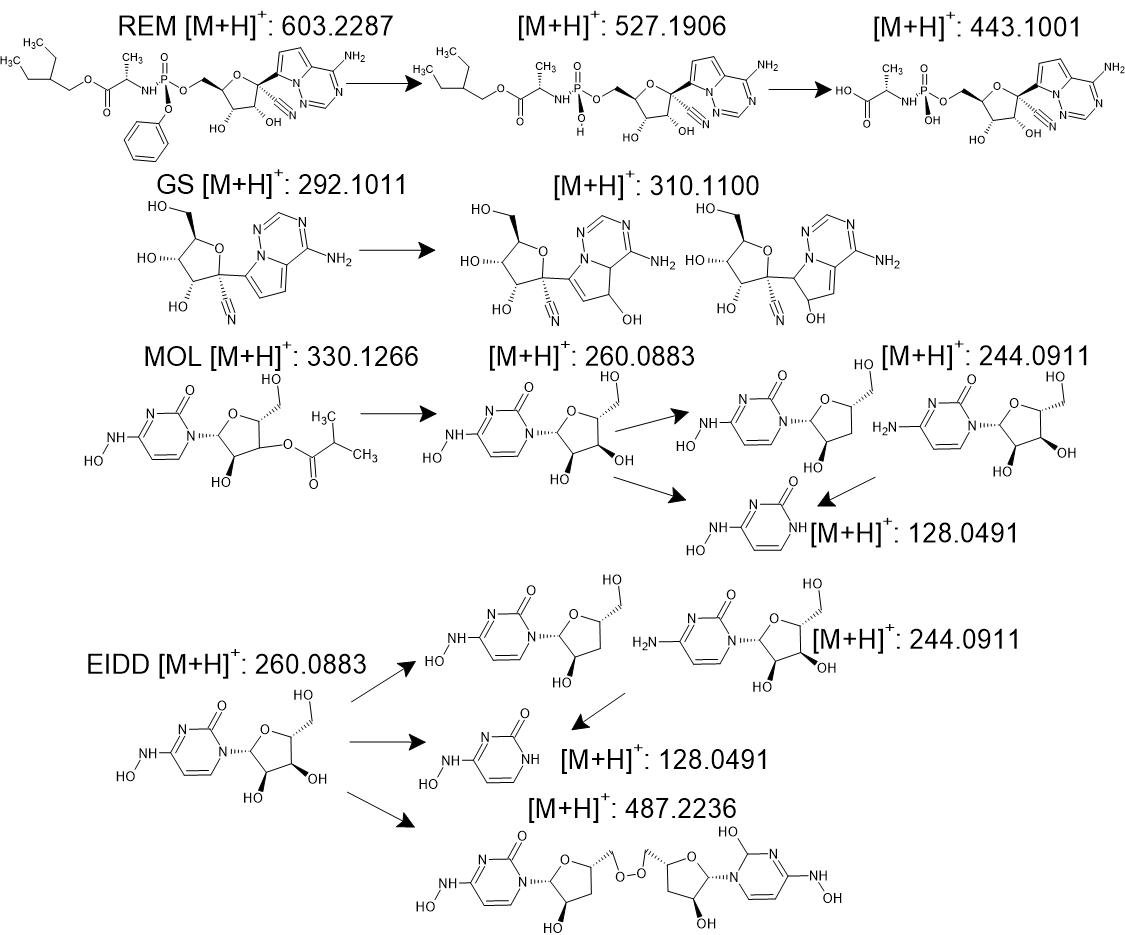


Figure A5 Investigation of the pH-dependent stability of REM, GS, MOL and EIDD, their predicted hydrolysis products and their proposed reaction scheme


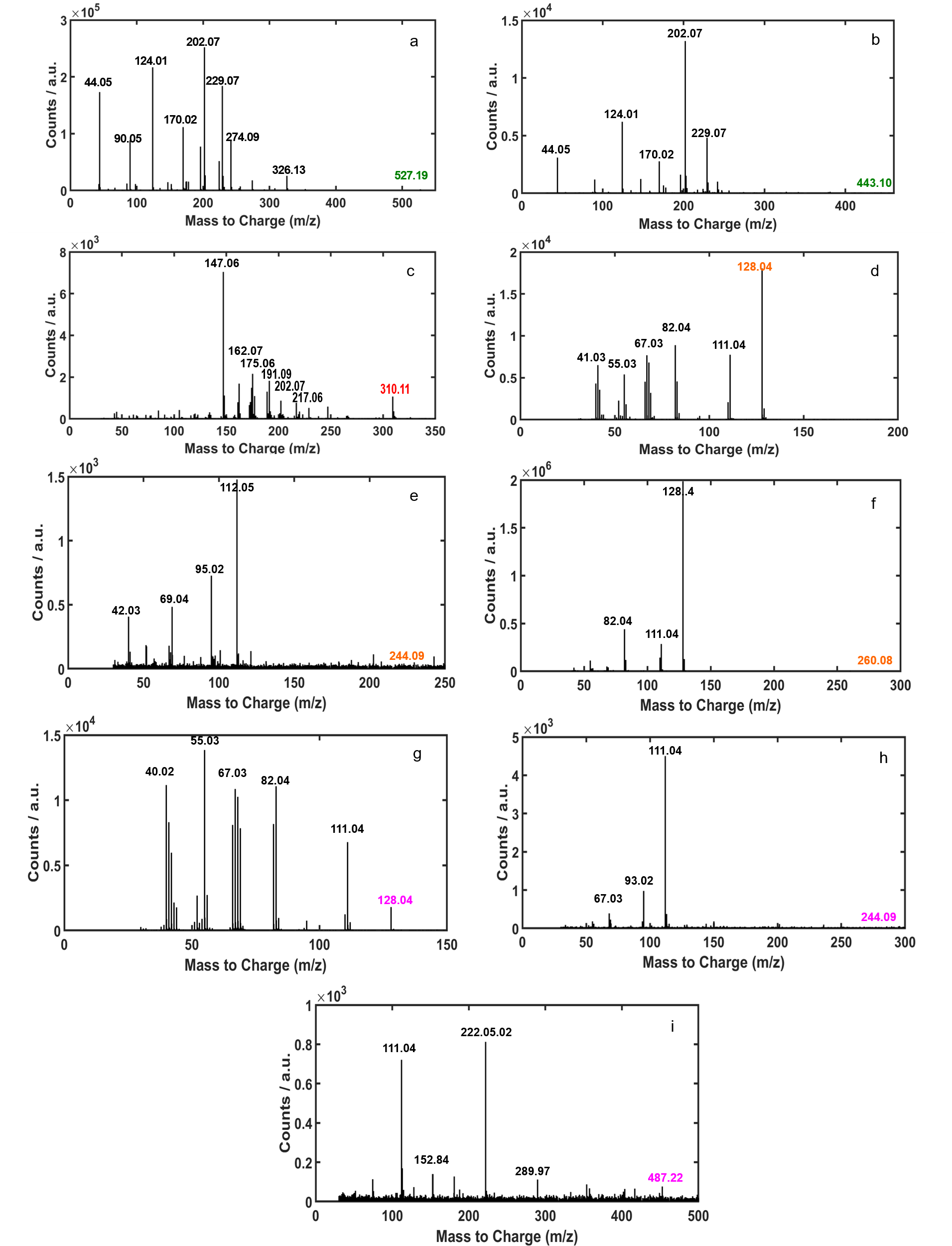


Figure A6 MS/MS spectra of hydrolysis products of REM TP 527.19 (a) REM TP 443.10 (b), GS TP 310.11 (c), MOL TP 128.04 (d), MOL TP 244.09 (e), MOL TP 260.08 (f), EIDD TP 128.04 (g), EIDD TP 244.09 (h) and EIDD TP 487.22 (i)
